# The antimicrobial activity and biocompatibility of a controlled gentamicin-releasing single-layer sol-gel coating on hydroxyapatite-coated titanium

**DOI:** 10.1302/0301-620X.103B3.BJJ-2020-0347.R1

**Published:** 2021-03-01

**Authors:** Tim Nichol, Jill Callaghan, Robert Townsend, Ian Stockley, Paul V. Hatton, Christine Le Maitre, Thomas John Smith, Robert Akid

**Affiliations:** 1 Biomolecular Sciences Research Centre, Sheffield Hallam University, Sheffield, UK; 2 School of Clinical Dentistry, University of Sheffield, Sheffield, UK; 3 Sheffield Teaching Hospitals NHS Foundation Trust, Northern General Hospital, Sheffield, UK; 4 Department of Materials, University of Manchester, Manchester, UK

**Keywords:** Sol-gel coating, Antimicrobial, Micro-CT, Controlled release, Uncemented prosthesis, Osseointegration

## Abstract

**Aims:**

The aim of this study was to develop a single-layer hybrid organic-inorganic sol-gel coating that is capable of a controlled antibiotic release for cementless hydroxyapatite (HA)-coated titanium orthopaedic prostheses.

**Methods:**

Coatings containing gentamicin at a concentration of 1.25% weight/volume (wt/vol), similar to that found in commercially available antibiotic-loaded bone cement, were prepared and tested in the laboratory for: kinetics of antibiotic release; activity against planktonic and biofilm bacterial cultures; biocompatibility with cultured mammalian cells; and physical bonding to the material (n = 3 in all tests). The sol-gel coatings and controls were then tested in vivo in a small animal healing model (four materials tested; n = 6 per material), and applied to the surface of commercially pure HA-coated titanium rods.

**Results:**

The coating released gentamicin at > 10 × minimum inhibitory concentration (MIC) for sensitive staphylococcal strains within one hour thereby potentially giving effective prophylaxis for arthroplasty surgery, and showed > 99% elution of the antibiotic within the coating after 48 hours. There was total eradication of both planktonic bacteria and established bacterial biofilms of a panel of clinically relevant staphylococci. Mesenchymal stem cells adhered to the coated surfaces and differentiated towards osteoblasts, depositing calcium and expressing the bone marker protein, osteopontin. In the in vivo small animal bone healing model, the antibiotic sol-gel coated titanium (Ti)/HA rod led to osseointegration equivalent to that of the conventional HA-coated surface.

**Conclusion:**

In this study we report a new sol-gel technology that can release gentamicin from a bioceramic-coated cementless arthroplasty material. In vitro, local gentamicin levels are in excess of what can be achieved by antibiotic-loaded bone cement. In vivo*,* bone healing in an animal model is not impaired. This, thus, represents a biomaterial modification that may have the potential to protect at-risk patients from implant-related deep infection.

Cite this article: *Bone Joint J* 2021;103-B(3):522–529.

## Introduction

The incidence of periprosthetic joint infection (PJI) is between 1% and 3% after primary arthroplasty and up to 15% after revision procedures.^1,2^ With the number of primary arthroplasties expected to increase to 1.26 million annually in the USA by 2030, significantly more patients will be affected by infection in the future.^3^ The interface between the prosthesis 'foreign-body' and the patient’s tissues presents a particular challenge in the prevention of infection. While prophylactic antibiotics are used to reduce the risk of PJI during primary surgery, once an infection becomes established on the prosthesis, antibiotics alone will not cure a PJI. This is because a biofilm has now become established and the only solution is radical surgery with appropriate antibiotic therapy. If, however, microorganisms entering the wound can be neutralized before a biofilm becomes established, the incidence of infection could be further reduced in both primary and revision surgery.

The local delivery of antibiotic provides higher concentrations of antibiotic at the site of infection than can be achieved by systemic therapy. While this delivery can be provided using antibiotic-loaded bone cement, it can obviously only be used with cemented components. While there is now an established trend for the use of cementless components at both primary and revision surgery,^2^ local antibiotic delivery is not therefore available for cementless arthroplasty. In order to provide a local antibiotic delivery system for cementless components, we developed a controlled-release antibiotic hybrid organic-inorganic sol-gel coating system for application onto a cementless implant material that is already coated with growth-promoting hydroxyapatite (HA).

Sol-gel systems are a group of materials which are prepared as a liquid sol that undergoes curing to produce a cross-linked matrix, which can be used as a coating. A range of these systems have been explored for biomedical applications, although there has been only one previous system tested as a drug-delivery coating for orthopaedic prostheses. This previous antibiotic-loaded sol-gel coating contained vancomycin and was tested on a titanium (Ti) alloy.^4,5^ It required a multilayer application and had limited opportunities for the tuning of its properties, since it only contained inorganic silica precursors. This study, using a rat *Staphylococcus aureus* osteomyelitis model, demonstrated antimicrobial activity in vivo when vancomycin was in the sol-gel.^5^

We have developed a hybrid sol-gel process (Supplementary Figure a) to prepare thin silica-based films with controlled pore size, hydrophobicity, and toughness for delivery of an antibiotic from the surface of the implant. The broad-spectrum antibiotic, gentamicin, was used as the antimicrobial agent as it is the antibiotic which is most frequently used in bone cement today. It is active against both Gram-negative and Gram-positive bacteria, which are responsible for the majority of PJIs. The coating was developed to provide the release of gentamicin during the time taken to undertake a routine primary hip or knee arthroplasty, typically between one and two hours. Furthermore, it was predicted that a rapid antibiotic-releasing coating of a few micrometres in thickness would avoid long-term low levels of antibiotic elution. Such low levels have been observed with bone cement, and have been implicated in the development of resistant microorganisms during prolonged infections.^6,7^ We have added the growth factor, bone morphogenetic protein-2 (BMP-2),^8,9^ to the coating to investigate any potential enhancement of bone-healing properties.

## Methods

Sol-gel coatings were prepared and cured at room temperature on 20 mm × 20 mm glass coverslips (for in vitro characterization) or HA-coated Ti cylinders (1 mm × 2 mm; for in vivo testing). Where stated, gentamicin and BMP-2 were incorporated into the coatings at 1.25% weight/volume (w/v) and 0.5 µg ml^-1^ to 2 µg ml^-1^, respectively. Where necessary, bone cement containing 1.25% (w/v) of gentamicin was used as a control material. Elution kinetics of gentamicin and BMP-2 were monitored analytically in vitro from coated glass samples. Antimicrobial activity of eluted gentamicin was confirmed against key pathogens in minimum inhibitory concentration (MIC) assays, as well as assays of biofilm formation and eradication. In vitro cytocompatibility testing and phenotypic assessment were performed using human mesenchymal stem cells (MSCs). In vivo biocompatibility analysis of the coatings on HA-coated Ti was performed in a rat healing model system. Full details of the methods can be found in the Supplementary Material, which also includes a completed ARRIVE checklist to show that we adhered to the ARRIVE guidelines.

### Statistical analysis

Statistical analyses were achieved using SPSS statistics software v23 (IBM, Armonk, New York, USA) and GraphPad Prism 6 software (GraphPad, San Diego, California, USA). For pull-out test analysis, the three coating types were compared using the Kruskal–Wallis test for non-parametric data. Statistical significance was set at p < 0.05.

## Results

### Elution characteristics of the coating

**Fig. 1:**
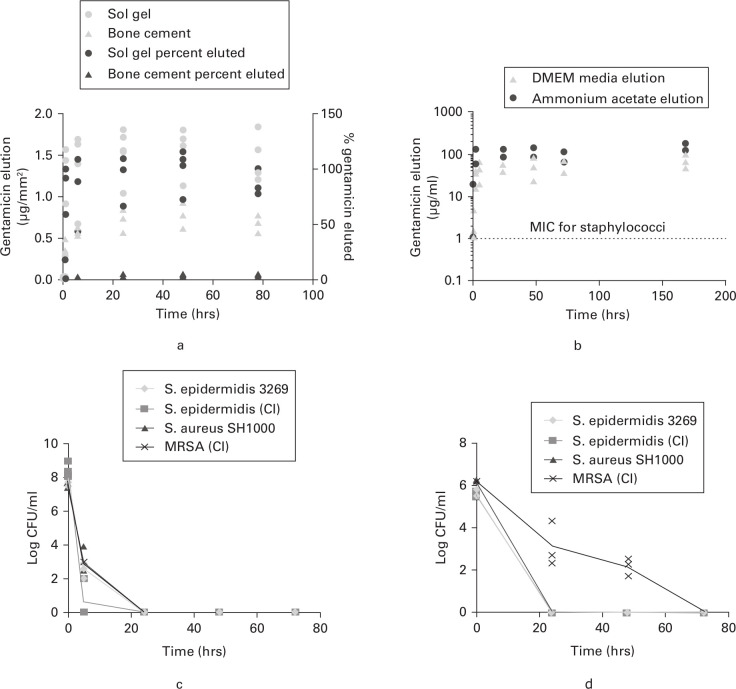
Elution and antimicrobial properties of the gentamicin sol-gel coating: a) Elution of gentamicin per mm^2^ surface area against time and % gentamicin eluted for sol-gel and gentamicin-loaded bone cement (n = 3). The 100% elution corresponded to 3.3 mg to 3.6 mg of antibiotic for bone cement samples (1.25% weight/weight (w/w)) and 625 µg for sol-gel (1.25% weight/volume (w/v)) samples. b) Semi-log plot showing elution of antibiotic from gentamicin sol-gel (1.25% gentamicin w/v) coated hydroxyapatite/titanium (HA/Ti) squares into 0.1M ammonium acetate buffer and Dulbecco's Modified Eagle Medium (DMEM) cell culture media (n = 2). The minimum inhibitory concentration (MIC) breakpoint for *Staphylococcus aureus* and *Staphylococcus epidermidis* (1 µg mL^-1^) is shown as a dashed red line. c) Time-kill curve for sol-gel coating (1.25% gentamicin w/v) against staphylococcal laboratory strains and clinical isolates (CI) from infected prostheses (n = 3). d) Time-kill curve for sol-gel coating (1.25% gentamicin w/v) against established biofilms of staphylococcal laboratory strains and CI from infected prostheses (n = 3). All data are plotted as individual values from replicate experiments. Where no bacteria were recovered, +1 was added to zero CFU ml^-1^ values before log transformation of the data. MRSA, methicillin-resistant *Staphylococcus aureus*.

The elution of gentamicin from coated samples was compared with that from samples of commercial gentamicin-loaded bone cement containing 1.25% gentamicin (w/v, weight/weight (w/w), respectively) monitored over a 78-hour period. Elution was monitored into ammonium acetate buffer and into Dulbecco's Modified Eagle Medium (DMEM) cell culture medium (to mimic the mix of salts and other small molecules that are typically found in vivo). The mean gentamicin concentrations eluted from the sol-gel into the ammonium acetate buffer reached a maximum of 1.557 µg mm^-2^ (SD 0.296) after 48 hours, representing > 99% of gentamicin contained within the coating. The mean amount of gentamicin eluted from bone cement reached a maximum of 0.775 µg mm^-2^ (SD 0.159) after 48 hours, representing only 4.98% of the original gentamicin contained within the cement (Figure 1a). Similar data were obtained with elution into DMEM medium (Figure 1b). The coating with gentamicin achieved the MIC for key target organisms (staphylococci) within one hour, i.e. within the operating time for a typical arthroplasty. Unlike the bone cement system, complete elution of the gentamicin from within the coating was observed within 48 hours, removing the possibility of problematic longer-term elution.

Sol-gel preparations containing gentamicin (1.25% w/v) and BMP-2 (0.5 to 2 µg ml^-1^) were coated onto HA/Ti squares and eluted into DMEM cell culture media, and the eluted BMP-2 concentration was measured by enzyme-linked immunosorbent assay (ELISA). After one week, concentrations ranged from 180 pg ml^-1^ to 256 pg ml^-1^, corresponding to only 0.2% to 0.7% of the BMP-2 eluting from the coating (data not shown).

### Antimicrobial properties of the gentamicin sol-gel coating

The gentamicin sol-gel coating was tested against a selection of clinical and laboratory strains of staphylococci. The antibiotic eluting from coated surfaces in a microtitre plate assay system gave a total kill of all clinical isolates and laboratory strains of *Staphylococcus epidermidis* and *S. aureus* within 24 hours (Figure 1c).

The gentamicin sol-gel coating was also tested against established biofilms from a selection of clinical and laboratory strains of staphylococci. Biofilms grown overnight on plastic pegs were submerged in fresh media, in wells of a 96-well plate containing the sol-gel coating. A total kill of the clinical isolate and laboratory strain of *S. epidermidis* and the laboratory strain *S. aureus* SH1000 was seen within 24 hours. The clinical isolate *S. aureus* strain showed a 3-log reduction between time 0 and 24 hours, and a total kill after 72 hours (Figure 1d)

### In vitro physical characterization of coating

The in vitro adhesive properties of the sol-gel on HA-coated Ti, HA-Ti wire coated with sol-gel + gentamicin (1.25% w/v), and HA-Ti wire coated with sol-gel + gentamicin (1.25% w/v) + BMP2 (2 µg ml^-1^) were assessed by a tensile pull-out test (Supplementary Figure b). All but two of nine samples that were tested snapped before pulling out of the bone cement (150 N to 220 N load). It should be noted that the cement into which the coated samples were embedded in these tests was antibiotic-free and used solely as a means to apply force to the outside of the coatings; there was no intention that the coatings should be embedded in cement during surgery. The samples that pulled out (~150 N) were not coated with sol-gel and failed at the interface between HA and Ti wire. There was no significant difference between the pull-out strengths of the samples (p = 0.875, Kruskal–Wallis test).

**Fig. 2:**
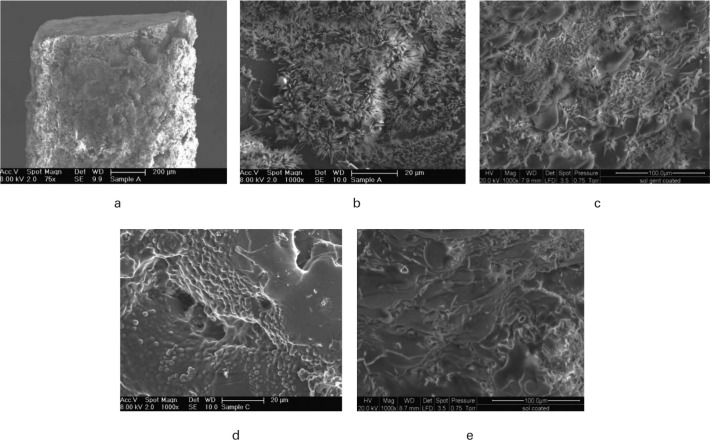
Scanning electron microscopy (SEM) images of titanium/hydroxyapatite (Ti/HA) rods (1 mm × 2 mm) coated with sol-gel + gentamicin (1.25% weight/volume (w/v)) or sol-gel only showing: a) cut rod section prior to gamma (γ) irradiation; b) coated sol-gel + gentamicin surface showing crystals prior to γ irradiation; c) coated sol-gel + gentamicin surface after γ irradiation; d) coated sol-gel only surface prior to γ irradiation; e) coated sol-gel only surface after γ irradiation.

The preparation of coated rods for implantation in the small animal healing model required the cutting of 2 mm lengths from coated HA-Ti wire. These sections were then gamma (γ)-irradiated prior to in vivo implantation. Samples were examined by scanning electron microscopy (SEM) before and after γ-irradiation to verify that the coating was still intact and that no delamination had occurred at the rod ends due to cutting. No delamination could be seen at the rod ends (Figure 2a), and crystals (gentamicin) could be seen on the gentamicin sol-gel coated surface (Figures 2b and c) and not on the sol-gel only coated rods (Figures 2d and e).

### In vitro osteogenesis of mesenchymal stem cells in contact with the coatings

**Fig. 3:**
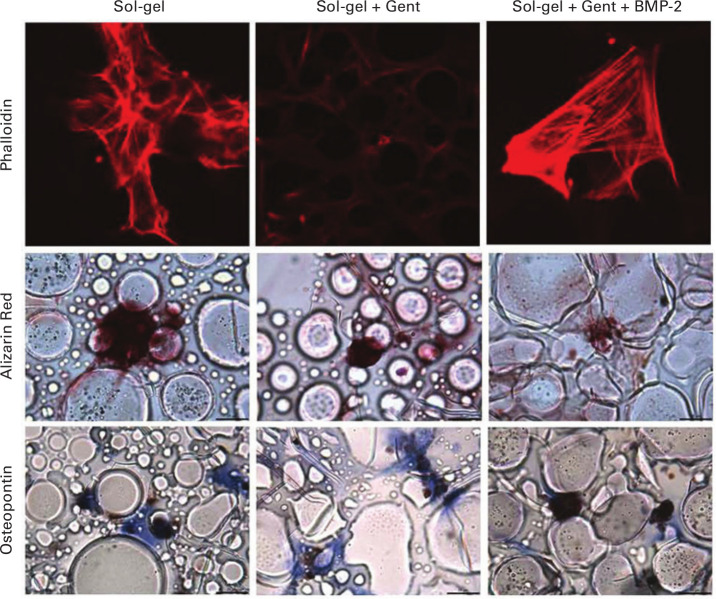
Fluorescence microscopy images showing mesenchymal stem cells (MSCs) on sol-gel coating. MSCs were cultured on the surface of coated coverslips for four weeks. Phalloidin stain shows a filamentous actin component of MSCs in red. Alizarin red stains calcium deposits (associated with bone formation) red, and osteopontin (stained brown) is an MSC metabolite indicating differentiation. In contrast, cells cultured on coverslips alone failed to produce any nodules. Scale bars = 20 µm. BMP-2, bone morphogenetic protein-2; Gent, gentamycin.

Rat MSCs seeded onto glass coverslips coated with sol-gel adhered and spread onto the surface of all sol-gel surfaces, regardless of the agents which were incorporated. Clear filamentous actin staining was seen, demonstrating cell spreading on the surface of sol-gel coated surfaces (Figure 3). Alizarin red staining demonstrated deposition of calcium in all samples (Figure 3), consistent with differentiation of the MSCs into osteoblast-like cells. In contrast, MSCs cultured on uncoated coverslips did not display any calcium deposition (data not shown). MSCs cultured on coverslips coated with all sol-gel preparations expressed osteopontin (a protein marker of osteoblast differentiation) regardless of the agents which were incorporated (Figure 3).

### In vivo osseointegration of implants coated with sol-gel

**Fig. 4:**
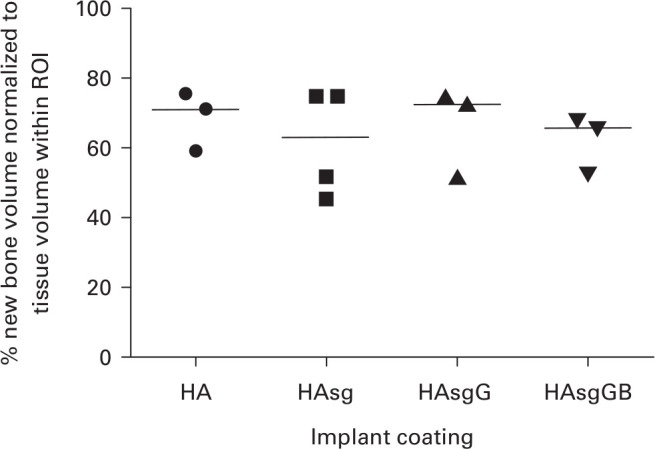
Micro-CT (µCT) analysis of the percentage new bone formation normalized to tissue volume within the region of interest (ROI), defined as new bone formation in contact with the surface of the implant. Four implant coatings were investigated: hydroxyapatite (HA) alone; HA + sol-gel coating (HAsg); HA + sol-gel containing gentamicin (HAsgG); and HA + sol-gel coating containing gentamicin and bone morphogenetic protein-2 (BMP-2) (HAsgGB). Data plotted as individual values from three or four samples from each test group subjected to µCT analysis; medians are shown as horizontal lines.

**Fig. 5:**
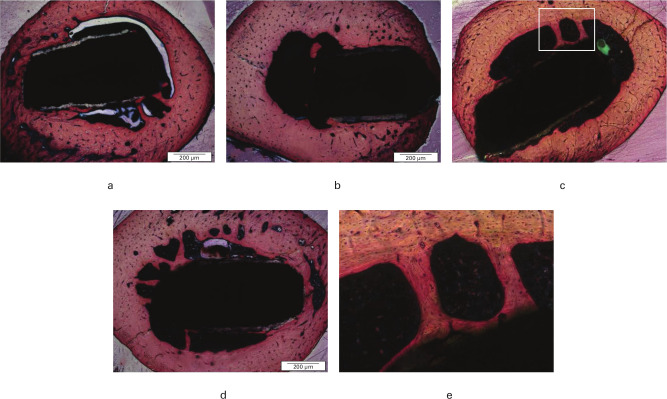
Typical sections of rat femur containing: a) hydroxyapatite-titanium (HA-Ti) implant (HA only); b) sol-gel + HA-Ti implant (HAsg); c) sol-gel + gentamicin (1.25% weight/volume (w/v)) + HA-Ti implant (HAsgG); d) sol-gel + gentamicin (1.25% w/v) + bone morphogenetic protein-2 (BMP-2) (2 µg ml^-1^) HA-Ti implant (HAsgGB); and e) highlighted section of HAsgG showing bone bridge formation. Sections were stained using Stevenel’s blue and Van Gieson’s stains, with new bone stained pink. Images are examples from analysis of six animals in each group and were taken on an Apero CS2 scanscope (Leica Biosystems, Milton Keynes, UK, set at 40× magnification).

When the implants were tested in the rat healing model, no clinical signs of infection were observed on any implant and all were immobile, suggesting that osseointegration had occurred. Micro-CT (µCT) analysis demonstrated that implants coated with sol-gel had comparable new bone volume to that of HA-coated Ti controls (Figure 4 and Supplementary Video 1). No histological differences were seen between any of the test groups and each implant was seen to be surrounded by normal lamellar bone in close contact with the surface of the implant. The sol-gel coated implants did not show any histological differences from standard HA-coated Ti wire implants (Figure 5). Vital bone marrow was also seen in contact with the implant between areas of bone contact. In all cases, bone formation could be seen in the medullary cavity close to the implant and spanning the distance between the implant and the cortical bone, forming bone bridges. The new bone involved both lamellar bone and, in places, less organized woven bone. There was no evidence of fibrous encapsulation or inflammatory response (Figure 5).

## Discussion

These results show that sol-gel coating has suitable properties to provide controlled local gentamicin prophylaxis for cementless orthopaedic prostheses, with bone healing properties that are comparable to an HA-coated surface currently in use. We found that the coating releases gentamicin in a controlled manner within a desirable time frame, with > 99% of the total antibiotic eluted within 48 hours. This time frame is compatible with effective prophylaxis during the peri- and postoperative period, to kill microorganisms resulting from surgical contamination and exclude the possibility of long-term elution.

When added, BMP-2 was retained within the coating and did not substantially elute. However, in vitro studies showed that BMP-2 was not required within the sol-gel coating to promote differentiation of MSCs, or required for effective in vivo bone healing.

Currently there are few options for local antibiotic delivery for cementless arthroplasty. A Defensive Antibacterial Coating (DAC) has been developed and trialled for use in hip and knee arthroplasties.^10,11^ This is a resorbable hydrogel, which can incorporate different antimicrobials and is applied by syringe during the operation. In vitro analysis of this coating has demonstrated elution of 99% after 96 hours of various antibiotics including vancomycin, gentamicin, and N-acetylcysteine. There was a reduction but not total eradication of *S. aureus* and *S. epidermidis* established biofilms in vitro over a 48-hour period.^12^ Clinical trials have shown that this system can reduce the rate of early surgical site infections and has not shown any adverse side effects.^10,11^ The physical nature of the hydrogel is somewhat different from the sol-gel coating that has been developed in this study. The more physically robust and thinner sol-gel may be less likely to be removed from the surface during insertion into bone. The DAC system does, however, enable tailoring of local antibiotic delivery to individual patients who undergo a two-stage revision procedure, and in a recent case-controlled study the results with respect to the control of infection were better in the coated group.^13^ This is something we plan to evaluate in due course, along with a potential use of the coating in single-stage revision surgery for PJI. The high concentration of eluted local antibiotic may well be very efficacious in this surgical scenario, in which high concentrations of antibiotic are essential for the success of single-stage surgery for PJI.

A silver-based coating, Agluna, has been developed by Accentus Medical (Oxford, UK), and uses Ti nanotubes containing silver ions to provide an antimicrobial coating for metal implants. Retrospective analysis has shown that rates of infection were reduced using this technology, particularly in two-stage revision procedures where antibiotic-loaded cement was also used.^14^ A similar silver-based coating, Modular Universal Tumour And Revision System (MUTARS; Implantcast GmbH, Buxtehude, Germany) uses galvanic deposition to provide a coating of elemental silver.^15^ This technology reduced infection in a rabbit model. Clinical studies have also shown a reduction in infection rates compared with non-coated implants. In practice, these coatings have mainly been applied to tumour megaprostheses for use in major skeletal reconstruction and not for routine orthopaedic implants. A recent animal test model of a silver-containing HA coating found that it significantly reduced the burden of methicillin-resistant *Staphylococcus aureus* (MRSA) infection, and that this effect was enhanced by introducing a vancomycin solution at the surgical site.^16^ A question still remains about the cytotoxicity of silver-based products, particularly long term.^17^

Synthes GmbH (Oberdorf, Switzerland) produce the Expert tibial nail PROtect, a tibial nail with a gentamicin coating that has shown promising results in preventing surgical site infections. This system uses a biodegradable polylactic acid coating for the release of gentamicin to provide antibiotic prophylaxis. Elution of 75% of gentamicin from the coating over 42 days was demonstrated in vitro, with a significant reduction in adhesion of *S. aureus* over 24 hours. In vivo elution studies showed similar results, although trace levels of gentamicin were detected after six weeks.^18^ However, this application is currently limited to the surgical treatment of tibial fractures and the technology has not been expanded for use with arthroplasties.^19^ A gentamicin-loaded ultra-high molecular weight polyethylene material enables local delivery of antibiotic, although its use is limited to spacers used in two-stage revision surgery.^20^

The sol-gels which have previously been shown to have potential as controlled release antimicrobial coatings used vancomycin, which provides no protection against infection caused by Gram-negative organisms.^4,5^ The single-layer hybrid sol-gel technology described in this paper targets a wider range of Gram-positive and Gram-negative organisms by using gentamicin. It is easy to apply and has the potential to adjust its hydrophobicity for elution of a wide range of therapeutic molecules, including antibiotics. Moreover, its antimicrobial and biocompatibility properties were demonstrated when it was applied to HA-coated implant material of the type widely used for promoting bone growth on uncemented prostheses. In vivo bone attachment was shown to be comparable to the HA-coated Ti surface of a type currently used in orthopaedic prostheses. In future, we propose to investigate the suitability of the coating for delivering a wide range of antibacterial and antifungal drugs, including combinations of antimicrobials, and application of the coating onto a range of materials used in orthopaedic surgery. We also propose to investigate a range of antibiotic loading levels and perform further in vivo testing, in particular to establish whether the coating can be modified quickly to kill biofilms as well as planktonic bacteria. In this way, it may be possible to design coatings which can protect against a wider range of organisms, facilitating a stratified or patient-specific approach.

**Take home message**

- There is a need for new systems to provide local antimicrobial prophylaxis for uncemented orthopaedic prostheses.

- Here we report a single-layer gentamicin-containing hybrid organic-inorganic coating capable of controlled release of active antibiotic.

- The coating is fully biocompatible with bone healing in a small animal in vivo system, suggesting promise for development as a coating for orthopaedic implants.
